# 2664. Patient Perspectives on Care Outcomes and Interactions with an Urban Emergency Department Health System for Mpox: A Quality Improvement Study

**DOI:** 10.1093/ofid/ofad500.2275

**Published:** 2023-11-27

**Authors:** Gaby Dashler, David Rudolph, Molly Brady, Luxi Qiao, Joyce Jones, Matthew M Hamill, Kelly Gebo, Mustapha O Saheed, Mun Heol, Nyah Johnson, Richard E Rothman, Yu-Hsiang Hsieh, Bhakti Hansoti

**Affiliations:** Johns Hopkins School of Medicine, Baltimore, Maryland; Johns Hopkins University School of Medicine, Baltimore, Maryland; Johns Hopkins University School of Medicine, Baltimore, Maryland; Johns Hopkins University School of Medicine, Baltimore, Maryland; Johns Hopkins University School of Medicine, Baltimore, Maryland; Johns Hopkins University School of Medicine, Baltimore, Maryland; Johns Hopkins, Baltimore, MD; Johns Hopkins, Baltimore, MD; Johns Hopkins University School of Medicine, Baltimore, Maryland; Johns Hopkins University School of Medicine, Baltimore, Maryland; Johns Hopkins University, Baltimore, Maryland; Johns Hopkins University, Baltimore, Maryland; Department of Emergency Medicine, Johns Hopkins University, Baltimore MD, Baltimore, Maryland

## Abstract

**Background:**

The Emergency Department (ED) is a safety net for vulnerable patients, and where almost 50% of patients with Mpox initially presented for care. We hypothesized that insufficient healthcare provider knowledge and expertise with Orthopoxvirus outbreaks, compounded by the social stigma experienced by patients at risk of acquiring Mpox, may impact the quality of care received by patients in this venue.

**Methods:**

All patients who received Mpox care in Johns Hopkins Health System Emergency Departments (N=4) were invited to participate in a post-discharge telephonic interview approximately six months after their index visit. Study staff interviewed and coded patients using a semi-structured interview script with three overarching themes “system,” “clinical,” and “individual” level barriers and facilitators to care delivery.

**Results:**

From July to November 2022, 47 patients received care for Mpox in the ED of which 13 agreed to participate; participants were mostly male (100%), living with concurrent HIV (54%) and identified as MSM (62%). Most patients were ultimately discharged from the ED (62%). The concern most often expressed by patients (n=7; 53%) was the lack of information regarding follow-up, isolation, and vaccine eligibility from the clinical staff, forcing patients to rely on the Internet and friends for information (n=5, 38%). Patients were also confused and frustrated about access to tecovirimat (n=4, 31%). Concerningly, experiences of stigma also played a significant role in patients' perception of access and quality of care from the ED (n=5, 38%).

**Figure:**
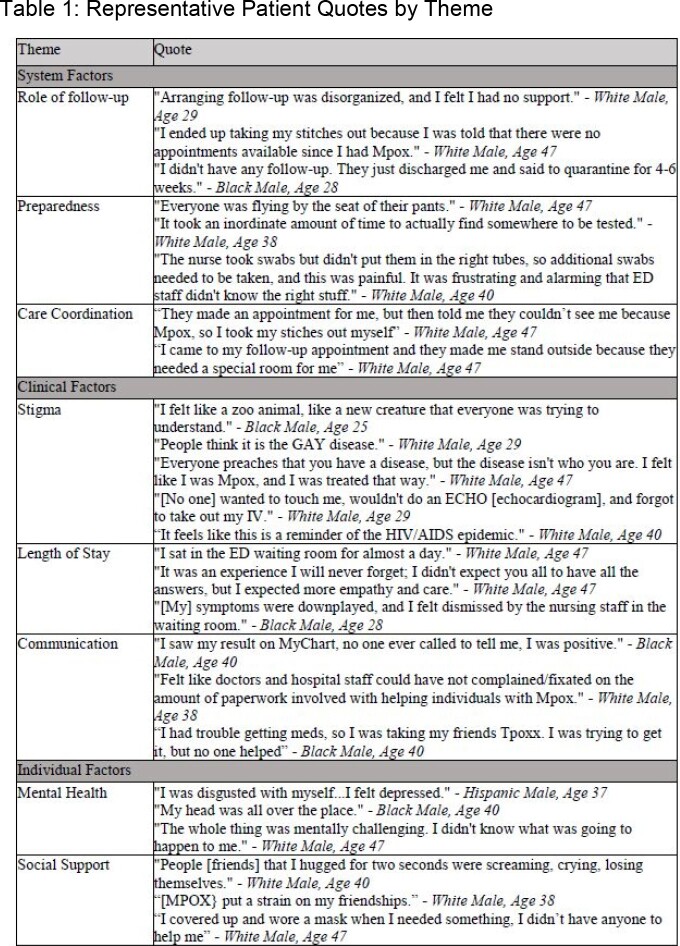

**Conclusion:**

Even though many patients with Mpox in the ED did not require hospitalization or treatment, the ED remained an important public health venue to help patients navigate this illness. Patients presenting to the ED often reported feeling stigmatized and uncertain about the next steps in their care post-diagnosis. Early, clear, concise public health messaging is critical to help improve provider and patient knowledge, as well as the patient experience. The ED is an important venue for risk communication and containment during an early outbreak response, especially for high-profile diseases that are under media scrutiny and where clinical uncertainty drive misinformation.

**Disclosures:**

**Matthew M. Hamill, MBChB, PhD**, Roche diagnostics: Honoraria **Kelly Gebo, MD, MPH**, Pfizer: Advisor/Consultant|Spark HealthCare: Advisor/Consultant **Richard E. Rothman, MD, PhD**, CEPHEID: Advisor/Consultant|Cepheid: Grant/Research Support|Danaher: Grant/Research Support

